# Breaking chains: IMAAVY redefines generalized myasthenia gravis treatment

**DOI:** 10.1097/MS9.0000000000003557

**Published:** 2025-07-16

**Authors:** Kamran Shahid, Syeda Rabiah Shahid, Maliha Khalid, Aminath Waafira

**Affiliations:** a Shaikh Khalifa Bin Zayed Al-Nahyan Medical And Dental College,Lahore, Pakistan; b Jinnah Sindh Medical University, Karachi, Pakistan; c The Maldives National University, Malé, Maldives

*To the Editor,*

In compliance with the TITAN guideline, this letter to the editor discusses the utilization, benefits, and prospects of the utilization of IMAAVY as a treatment for generalized Myasthenia Gravis^[^1^]^. On 30 April 2025, the U.S. Food and Drug Administration (FDA) approved IMAAVY™ (nipocalimab-aahu, FcRn-blocking monoclonal antibody) for the treatment of generalized Myasthenia Gravis in adult and pediatric populations older than the age of 12^[^2^]^; marking a momentous breakthrough in the management of this debilitating disorder.

Myasthenia gravis is a chronic autoimmune neuromuscular disease—with a global prevalence estimated to be between 150 and 250 cases per million people, commonly affecting young women and older men—characterized by weakness in voluntary muscles, including those used for movement, breathing, and swallowing^[^3^]^. This incapacitating disease stems from antibodies blocking acetylcholine receptors (AchR) at the neuromuscular junction, preventing muscle contraction—a simple yet paradoxically complex condition. Common symptoms include drooping eyelids, double vision, facial changes, difficulty swallowing or speaking, and limb weakness. A myasthenic crisis, often triggered by infection or stress, may require emergency care. Treatments with anticholinesterase agents (e.g., pyridostigmine), thymectomy, immunosuppressants, complement inhibitors (e.g., eculizumab), and short-term therapies like plasmapheresis and IVIG have been shown to improve muscle strength and function. Yet, no cure is currently available^[^3^]^.

IMAAVY, the first of its kind, is a fully human FcRn-blocking monoclonal antibody (mAb) that targets highly specific binding to FcRn, both intracellularly and extracellularly. Engineered for durability, high affinity, and precision, IMAAVY has proven to be effective in reducing pathogenic circulating IgG, anti-AChR and anti-MuSK autoantibody levels. Its mechanism supports long-lasting immunomodulation with minimal adverse immune response to the drug owing to its entirely human-derived property^[^4^]^. Thereby, offering a novel therapeutic approach for managing this autoimmune disorder.

This FDA approval follows positive outcomes seen in various clinical trials, one of them being the ongoing Vivacity-MG3 study: a phase 3, randomized, double-blind, placebo-controlled, phase 3 study^[^2^]^. The trial demonstrated a rapid improvement in the measured outcome of MG- ADL within the very first week, with the recovery being maintained throughout the course of the study^[^5^]^. Moreover, the simultaneously ongoing phase 2/3 Vibrance-MG study directed at pediatric populations revealed a notable reduction in the levels of immunoglobulin G(IgG) over a period of 24 weeks with several participants experiencing minimal symptom expression as well^[^6^]^.

Although IMAAVY was very well tolerated and no new safety signals were identified, some of the common adverse effects of the drug are an increased risk of infections and hypersensitivity reactions. Infusion-related side effects are also possible, hence why the drug is contraindicated for patients with an allergy to nipocalimab-aahu or any of Imaavy’s ingredients^[^2,5^]^.

The FDA’s approval of IMAAVY represents a breakthrough toward providing long-lasting disease control to patients afflicted with generalized myasthenia gravis. It has the capability of revolutionizing our approach regarding the management of myasthenia, however further research is needed to address its long-term concerns as well as acquire more detailed information about its administration and effectiveness.

Unlike earlier commentaries of IMAAVY’s approval being found on blogs or news articles, this letter aims to provide a brief and concise peer-reviewed overview of its approval, trial outcomes, and implications within the context of a medical journal.

## Data Availability

No data were generated for this manuscript.
